# Modern Sociology

**Published:** 1900-02-10

**Authors:** 


### Modern Sociology.

### THE KNIGHTS HOSPITALLERS.

As the Knights of St. John of Jerusalem, commonly called the Knights Hospitallers, may claim to be regarded as the first army medical corps, an account of them may not be uninteresting at the present time. The order found its origin, however, rather in the management of a guest-house than in a hospital. The first home of the Order was in Jerusalem, and its object was to afford a temporary home to the pilgrims who visited the Holy Sepulchre, and were then subjected to scorn and indignity by the Saracen conquerors of the Holy Land. It was in the eleventh century that some merchants of Amalfi purchased permission from the lords of Syria to establish near the Holy Sepulchre a place of refuge for visitors to Jerusalem. This was put under the charge of an abbot and monks of the Benedictine Order. Near by was a convent where a sisterhood of kindly nuns received and cared for female pilgrims.

It was in 1099, when Jerusalem was invested by the Crusaders, that the more strictly hospital work (in the modern sense) of the Hospitallers began. The Hospital of St. John was at that time presided over by Gerhard, a native of Provence, and a man whose ideas of charity were far in advance of his time. To him the Christian and the infidel were all equal when they were wounded or sick. Crusader and Moslem alike shared in the attentions of him and his monks. After the taking of Jerusalem by the Crusaders, Gerhard and his monks devoted themselves more specially to the care of the sick, and though still under the law of the Church and the rule of the Benedictine Abbot, they formed themselves into a separate brotherhood. They assumed what became their distinctive dress—a black mantle, with a white cross of eight points upon the left breast, what we now call the Maltese cross. Godfrey de Bouillon and his successors gave great donations to the order; so, though it began with a vow of poverty, it soon became rich and powerful.

It was not till the Christian kingdom of Jerusalem was pressed hard by the Saracens that the Hospitallers changed their views of their mission, and joined the ranks of Christian warriors; but with this change came a great accession to their ranks, and a complete change in their character. The Order was now divided into three classes: Knights of noble birth, priests and almoners, and servants. A further division was made according to the countries the knights came from—Provence, Auvergne, France, Italy, Arragon, Germany, and Anglo-Bavaria. All through the thirteenth century the Hospitallers fought bravely for the Holy Land. In the war against the infidel they were supported by the kindred body of the Knight Templars; but between the two orders there was a jealousy that hindered their success. In 1291, the Sultan of Egypt laid siege to Acre, by that time the last Christian stronghold in Palestine. Both of the Christian military orders assisted at its defence. Of these every Templar, including the Grand Master of the Order, was killed, and only seven of the Hospitallers escaped.

They landed in Limasol, in Cyprus, and from there the Grand Master tried to organise a new crusade; but the enthusiasm was worn out, and the only conquest made by the Hospitallers was that of the island of Rhodes, where for 200 years they resisted the power of the all-conquering Turk, and harassed all Turkish and Egyptian ships. To us who know the Turk only as a poor and effete power, kept in his place in Europe rather by the jealousy of Christian nations than by his own strength, this may seem a small feat; but in those days, when the impetus given by Mahomet had not wholly faded out, and religious fervour was supported by the merciless cruelty of Orientals, it required endless courage and watchfulness to be, as the Hopitallers were, the bulwark of Europe and Christendom against the infidel. Attacked in their island citadel again and again, they held it against heavy odds for 200 years. But in 1522 they were forced to surrender to the Sultan Solyman II. Even then they were able to command honourable terms of capitulation, and it was at this time that they assumed the famous red-cross flag—a white banner with a red cross upon it, on each arm of which was embroidered the letters F.E.R.T., which stood for "*Fortitudine Equites Rhodum Tenuent*"—"By courage the Knights held Rhodes."

For more than seven years the knights had no special home and stronghold, though they had rich houses in every country in Europe; but on March 4th, 1530, the Emperor Charles V. granted to them the ownership of all the castles, fortresses, and isles of Malta, Gozo, and Tripolis. Thenceforward the Hospitallers were the "Knights of Malta." At the time they obtained possession of the island, Malta had only 12,000 inhabitants, and Gozo 5,000, all sunk in the deepest poverty. Under the protection of the Knights the inhabitants began to cultivate the land once more, and regained a long-lost prosperity. The Knights fortified the island, and held it against the repeated attacks of the Turks. In 1666 the Grand Master of the time, La Vallette, founded the city that bears his name. The fortifications of Valletta still bear witness to the energy, ingenuity, and wealth of the men who built them. It was not from the resources of Malta that the funds were obtained to build these walls; but contributions came from the richest countries of Europe to strengthen this last bulwark against the Turk. But as the Turkish power decayed, the Knights of Malta degenerated. Secure in their island fortress, no longer in constant expectation of attack from the infidel, they lost their hardihood, and became effete and useless. It was Bonaparte who destroyed them, though it seems probable that the connivance of the French knights, as well as the general weakness of the whole Order, contributed to their downfall. In 1798 Bonaparte demanded permission for the whole French fleet to enter the ports of Malta for water. The Grand Master said that only two, or at most four, ships could be permitted to enter at a time. This was excuse enough for Napoleon, who ordered an immediate landing on the Maltese coast. Within a week the Grand Master had capitulated, and on June 17th, 1798, he left Malta for ever.

This was the end of the Knights Hospitallers as a military power. As a religious order they still survive, and have a small settlement in the city of Ferrara. But their best memorial survives in the St. John Ambulance Associations, which are named after the once-famous order; the red cross flag that waves over the hospital on the battle-field; and the Royal Red Cross, which is the reward of those who have won distinction in nursing the wounded. Not in their wealth, nor even in their prowess, but in their having in a barbarous age conceived and carried out a noble and humane idea lies the real and permanent glory of the Knights Hospitallers.

